# Antiseptics leave the Clinic—The Introduction of (Puerperal) Prophylaxis in Austrian Midwifery Education (1870s–1880s)

**DOI:** 10.1093/shm/hkab097

**Published:** 2021-10-14

**Authors:** Marina Hilber

**Keywords:** antisepsis, midwifery, knowledge transfer, Austrian Empire, nineteenth century

## Abstract

By introducing compulsory antiseptic measures in 1881, the Austrian Empire became a trendsetter in European midwifery legislation. Starting with the focus on puerperal infection in the 1870s, this article investigates the process from the first proposal of antiseptic regimes within a clinical setting to the dissemination of antiseptic knowledge among the midwifery profession. Competition between the leading medical men throughout the Austrian territories played a major role and influenced the way in which antiseptic measures were propagated. The article identifies the antiseptic collectives active at the leading universities of Prague and Vienna. As practical instruction during midwifery education was not regarded as sufficient, the late 1870s and 1880s saw the emergence of several instruction textbooks. Changing birth attendance routines and the innovative materials that entered midwifery practice are explored and discussed, based on these manuals, alongside evidence of midwives’ reactions as published in the Austrian Midwifery Newspaper.

In October 1887 the midwife Mrs B.B. from Neusohl (today: Banská Bystrica/Slovakia) wrote a letter to the newly established Austrian Midwifery Newspaper inquiring whether she was really obliged to carry disinfectant solution along to all her domiciliary visits. A reply to her letter was published in the advice column of the third issue confirming that, according to the midwifery regulations, a bottle of two per cent carbolic acid should always be in her bag. The editors even provided a detailed description of the process of preparing an effective yet non-toxic carbolic acid solution.^1^ In the fourth issue from mid-November 1887 Mrs M.M., a midwife from Weissenkirchen in Lower Austria, complained that the local chemist refused to sell concentrated carbolic acid to her. The editors explained that there was a law prohibiting the purchase of the substance by lay people, however, she could ask the local physician for a prescription and would thus be able to obtain the antiseptic agent.^2^ In the following issue Mrs M.J., a midwife from Vienna, wanted to know how she could best prevent her hands from cracking when using antiseptic fluids. Again, the editors provided qualified suggestions, advising that, besides rinsing her hands with tepid water, drying them thoroughly and keeping them warm, she could also make use of glycerine to soften her skin.^3^

These letters from midwives practising in the Austrian Empire in the late nineteenth century touch upon an aspect of midwifery practice that was still a relative novelty. The routines of antiseptic obstetric practice propagated since the Austrian countries’ 1881 Revised Midwives’ Instruction were obviously not yet fully embedded. Midwives often struggled to adapt to the new routines, lacking support and instruction from the experts. This article focuses on the introduction of antiseptic routines to birth attendance—the most fundamental reform in midwifery practice since the establishment of midwifery courses in the mid-eighteenth century. Of interest here are the academic debates among the expert commissions, networks and alliances of leading obstetricians within the Austrian Empire, and these experts’ strategies of initiating and integrating midwives into their new scheme.

The article starts with a general look at the state of midwifery education and practice in the Austrian Empire in the second half of the nineteenth century, and introduces the framework of the set of regulations known as the Midwives’ Instruction. A brief literature review looks at recent research into the effect of state efforts to control maternal mortality elsewhere in Europe. The article then goes on to examine the rise of germ theory^4^ and antiseptic understanding^5^ among the medical elite in the Austrian Empire, and the process by which it was agreed that this knowledge would have to be disseminated to the midwifery profession.

In looking at the adoption of new methods in obstetrics, questions of knowledge transfer are raised. The second part of this paper therefore asks how knowledge was conveyed, scrutinising the media of dissemination. What information and which techniques of antiseptic prophylaxis did medical men find suitable for transfer outside the clinical context? Was the knowledge transfer purely top-down, or did midwives themselves have any agency in their appropriation of knowledge? Given the medical experts’ viewpoint on the intellectual capacities of women, issues of gender can clearly be seen in the way that knowledge was disseminated to the midwives. Instructional information, it was thought, had to be adapted and simplified without risking inaccuracy.^6^

In the third part, the impact of changing routines and the introduction of new materials are discussed. The study reconstructs the process of translating knowledge into practice; of adopting and implementing changed routines in home births at a time when there was still no certainty about the most effective means of prophylaxis. Special focus is laid on the struggles midwives experienced when confronted with a new regimen of cleanliness. The study explores how midwives reacted to the changing routines, and documents some of the concerns held by those entrusted with the application of antiseptic measures.

The study mainly draws on archival sources consisting of official reports, minutes from the meetings of the *Oberster Sanitätsrat* (Supreme Health Council), legislative material, instructional texts as well as articles and reader’s comments in the contemporary medical and midwifery press. A limitation of this study, due to the constraints of historical statistics for the Austrian Empire, is the unavailability of maternal mortality data for the 1870s and 1880s. This is because puerperal fever only formed part of post-mortem protocols from 1895 onwards.^7^

By focusing on the eventful time prior to and following the 1881 *Revidirte Hebammen-Instruction* (Revised Midwives’ Instruction), this article relies on a qualitative approach and attempts to disentangle the ‘chaos of conflicting opinions’ that Michael Worboys observed to exist.^8^ Thus, the study contributes to the history of midwifery education and obstetrics and the history of puerperal prophylaxis, as well as the social history of medical knowledge in the realm of the Austrian Empire.^9^

Despite early attempts, midwifery education in the Austrian Empire was far from standardised in the nineteenth century.^10^ Academic freedom, granted after the 1848 revolution, had created a fragmented system where each and every crown land developed its own educational approach. Midwifery instructors were not only free to choose the content taught but also the duration of courses, varying from two, later four months in Prague, to six months in Innsbruck or eight months in Czernowitz. Thus, the knowledge base among midwives was highly heterogeneous throughout the Empire, in terms of both theoretical knowledge and practical skills acquired throughout their training. Midwives had to report the uptake of their professional practice to the medical authorities and were to be regularly inspected by the district physicians. Local priests exercised further control as they recorded the population development in their parish registers, and midwives were compelled to notify the authorities of every birth they attended. In the case of irregularities, such as an accumulation of childbed-fever-related deaths, the medical authorities would be called in.^11^

Most trained and officially licenced midwives were educated on behalf of the local municipalities, who paid for the course fees and provided the trainees with board and lodging for their period of training in town. In exchange, these women committed themselves to assisting all births in their communities, especially in the homes of the poor. For their willingness to attend the local poor, community midwives were entitled to a small annual lump sum. For all other births attended the midwife could charge a fee, but in many cases she was paid not in money but in kind, such as with wood, lard or eggs. The assistance of a midwife was still widely considered an act of benevolence and therefore many families did not feel obliged to pay anything at all. Especially in rural areas, therefore, many midwives lived in dire economic and social conditions. The plea to legally secure at least a minimal regular income became louder throughout the second half of the nineteenth century, especially once the activities of the newly established midwifery societies gained momentum.^12^ Immediate support was granted by Austria’s leading obstetricians, who saw adequate wages as the key to improving the quality of obstetric care.^13^

Although the total numbers of qualified midwives rose throughout the century, increasing population numbers impeded political efforts to improve the availability of midwifery services in the Austrian Empire. Between 1848 and 1900 the ratio had even dropped from 197 to 194 midwives per 10,000 births (live and dead) as an average throughout the Empire. Yet there were major differences between the respective crown lands. While Tyrol and Vorarlberg, as well as Upper Austria, displayed a relatively good situation recording more than 300 midwives per 10,000 births, regions with less developed medical infrastructure such as Galicia, Silesia or Dalmatia registered devastatingly low numbers far below 100 midwives per 10,000 births.^14^

For the better part of the nineteenth century, the 1808 Midwives’ Instruction regulated the daily practice of qualified midwives in the Austrian Empire. It touched upon aspects of availability (24-hour on-call services), sobriety, and moral demeanour, and also outlined midwives’ religious duties should an emergency baptism become necessary. Medical issues were vaguely settled, the midwives being compelled to treat only one parturient woman at a time, to call a physician in cases of emergency, and to stay with the woman for a certain time after birth in order to control haemorrhage.^15^ The 1874 Midwives’ Instruction did not change profoundly; the most important aspect of revision was that midwives had to ask for the parents’ permission before administering an emergency baptism.^16^ When a revised Midwives’ Instruction was issued only seven years later, in 1881, this must have happened for a compelling reason. In this article it is assumed that the rapidly increasing focus on puerperal infection in the 1870s triggered the re-evaluation of midwives’ duties.

In the context of the medicalisation and professionalisation of midwifery services, several researchers have scrutinised the effect of state regulations aimed at addressing the ever increasing numbers of maternal and infant fatalities throughout the nineteenth century. With the rise of social historical perspectives in medical history since the 1980s, ground-breaking studies using quantitative approaches to examine maternal mortality and its social and medical implications have been published. Ulf Högberg was one of the first to argue that improving the quality and supply of obstetric provisions had an immediate effect on reducing maternal mortality, not only in the clinical setting but also in home deliveries. According to his study, antiseptic measures played an important role.^17^ Based on the example of Britain, where trained and certified midwives were not available until the beginning of the twentieth century, Irvine Loudon contributed the assumption that it was not only the availability of qualified midwives that accounted for the decrease in maternal mortality, but also a decline in the virulence of the streptococcus strain that caused most of the infections.^18^ However, he agreed that the proper use of antiseptics correlated with the quality of educational standards.^19^ This was also acknowledged by Anne Løkke’s study on the ‘Antiseptic Transformation’ of midwifery services in Denmark.^20^ Comparative studies using mainly demographic data in a trans-regional focus further support this notion.^21^ Stephan Curtis was able to add yet another perspective as he analysed the role patients played in reducing maternal mortality. He could prove that although the number of qualified midwives in Sweden increased since the 1880s, parturient women in some regions were still reluctant to call upon the ‘new’ midwives with their modern techniques of antisepsis. Women in rural areas especially stuck rather to suppliers of traditional obstetric care.^22^

## Antiseptic Collectives

Most childbed-related deaths during the nineteenth century were attributed to post-partum infection, widely known as childbed or puerperal fever.^23^ The disease was not only described as a dangerous and widespread illness but also estimated to cause even more fatalities than smallpox or cholera.^24^ Around the mid-1870s there was still disagreement about the aetiology of the disease, and a generally accepted characterisation of its cause(s) was still pending. The following section first reconstructs the most prominent ideas on possible causes discussed by the leading Austrian obstetricians and second elaborates on the circumstances that led to the introduction of compulsory antiseptic measures into Austrian midwifery practice.

Almost thirty years after Ignaz Semmelweis (1818–65) had first proposed his theory of a single necessary cause of puerperal infection, this was still contested among the leading experts. Also contested, however, was the long list of thirty unrelated causes (i.e. milk secretion, chilling, wrong diet, missing ventilation, crowded hospital wards), compiled by Carl Braun (1822–91), Semmelweis’ successor as an assistant professor in Vienna and one of his most ardent opponents. Braun reduced his extensive list in later life, ultimately emphasising the importance of ventilation to prevent miasmic infection which he described as ‘toxic dried vegetable organisms conveyed through the air’.^25^ However, while concepts of miasma theory still prevailed in many places, especially influencing hospital architecture and ventilation technology, some of the leading Austrian obstetricians gradually came to accept Semmelweis' alternative theory. The aspiring Prague obstetrician Ludwig Kleinwächter (1839–1906), though avoiding any reference to Semmelweis, summarised the prevailing views on the topic in his 1877 obstetric textbook. He understood puerperal fever as a blood disease caused by decaying organic substances. These could either be amniotic or placental residues, or could enter from the outside via contaminated instruments or the examining hands of doctors and midwives. The decomposing matter would find its way into the bloodstream via birth wounds, thus leading to sepsis. However, the reason for the harmfulness of these substances had not yet been discovered. Theories about the influence of the tiniest and ‘lowest organized structures’, identified as bacteria or micrococci in contemporary debates, still awaited scientific confirmation. Nonetheless, Ludwig Kleinwächter concluded that ‘puerperal fever should be considered a contagious disease’.^26^

While the aetiology gave reason for disagreement and room for further research, strict hygienic measures had entered most maternity clinics throughout the 1870s. This was, however, not due to a rediscovery of Semmelweis’ advocacy for washing with chlorinated lime.^27^ It was, in fact, the elaborate method of wound treatment introduced to surgery by Joseph Lister in 1867 that triggered a new evaluation of disease prophylaxis in the field of obstetrics.^28^ The Bohemian obstetrician August Breisky (1832–89) was one of the most committed proponents of antiseptic disinfection in the Austrian Empire. After having succeeded in reducing mortality at the Berne Maternity Clinic, where he had become full professor in 1867, he became a fervent advocate of puerperal prophylaxis on returning to Prague as Professor of Gynaecology and Obstetrics in 1875.^29^ In alliance with Ferdinand Ritter von Weber-Ebenhof (1819–93), midwifery instructor at the University of Prague and father-in-law of the aforementioned Ludwig Kleinwächter, Breisky successfully reduced mortality in the Prague Maternity Clinic from 2 to 0.5 per cent by 1879.^30^ The success of the antiseptic measures was not only reflected by a noticeable reduction in puerperal mortality rates, but also by a reduction in morbidity among lying-in women. Within a year, the midwifery clinic in Prague run by Weber-Ebenhof documented a gradual reduction in cases of post-partum infection from 16 to 5 per cent.^31^

As soon as antisepsis had been embedded into obstetric routines in the clinical environment and mortality numbers were seen to be decreasing, doctors turned their attention to further improvements. Clinical experimentation on the therapeutic and prophylactic application of various disinfectant substances dominated much of the following decade.^32^ Yet, another challenge in optimising obstetric care lay beyond clinical applications. The question of whether and how antiseptic measures were to be introduced to regular midwifery practice first arose in July 1877, when an anonymous appeal for a revision of the 1874 Austrian Midwives’ Instruction was made.^33^ The following section investigates the reactions of the ministries and expert commissions involved in the negotiations, highlighting not only the heated scientific debates between the country’s leading obstetricians but also the dominance that Viennese medical elites exerted over their rivals in other parts of the Empire.

The leading role of Vienna was defined by its university, which was the largest and most prestigious in the Austrian Empire. A chair at the University of Vienna was the culmination of an Austrian medical career, but also the end of the line. Many of the professors took over at an advanced age and tended to stay until retirement or death. The specific appointment practice thus led to an over-aged professorate that was less open to innovation.^34^ This can be seen, for example, in Theodor Billroth’s (1829–94) scepticism towards bacteriology. Vienna’s most prominent surgeon criticised Pasteur and showed reluctance when it came to antiseptic measures, leaving the examination of scientific innovation, such as the evaluation of Lister’s wound dressing, to his assistants.^35^ In the case of failure, his reputation would not have been harmed. However, the dominance of the Viennese professors manifested itself not only in academia, but also on a political level. The influential Supreme Medical Council, for example, was composed of the leading Viennese experts. In this way, their influence extended far beyond the capital.

Although none of the established members of the medical councils had so far seen the need for alteration or augmentation of the existing Midwives’ Instruction, the proposed fight against puerperal fever in home births and the prevention of numerous maternal deaths seemed an intriguing perspective to the medical and political representatives. The appeal received immediate attention, and the Ministry for Cultus [Religious Affairs] and Education informed the Ministry of the Interior that ‘[t]he wish for the issuing of a comprehensive instruction, providing for all contingencies of midwifery practice was made by a competent source’.^36^ It can hardly be a coincidence that the proposal was made at the same time that one of the rare full professorships became available in the Austrian Empire.^37^

Following the unexpected death of Virgil von Mayrhofen (1815–77) in June 1877, four aspiring Austrian obstetricians hoped for an appointment at the University of Innsbruck.^38^ Three of the four contestants could have been the ‘competent source’, as they had previously worked on aspects of puerperal prophylaxis. While Karl Mayrhofer (1837–82)^39^ and Karl Freiherr von Rokitansky (1839–98)^40^ had both engaged in research on the aetiology of puerperal fever,^41^ Ludwig Kleinwächter had shown a more pragmatic approach as he promoted the practical implementation of antiseptic measures. Although he did not invent a new system but rather stuck to the regulations proposed in Joseph Amann’s *Klinik der Wochenbettkrankheiten* (Clinic of Puerperal Diseases),^42^ his 1877 obstetric textbook was, in fact, one of the first in the Austrian Empire to include a detailed chapter on puerperal prophylaxis and disinfection guidelines for medical students and practitioners, going beyond basic aspects of cleanliness.^43^ As the medical faculty at the University of Innsbruck was still more interested in teaching skills than scientific output, Kleinwächter was their first choice.^44^

Even before Kleinwächter was officially appointed in August 1877, the Ministry for Cultus and Education had invited him to draft a new midwives’ instruction. Kleinwächter reviewed the existing 1874 Midwives’ Instruction and drafted substantial additional details in regards to disinfection. The result was approved by the Tyrolean Medical Council and forwarded to the Ministry in January 1878.^45^ ‘According to modern scientific experience’, Kleinwächter argued,

childbed fever has to be considered a contagious disease which can be passed on to healthy puerpera by instruments and devices such as catheters, enema syringes, sponges, etc. as well as through filthy hands contaminated by touching women suffering from childbed fever. It is therefore crucial to instruct midwives to strictly adhere to the disinfection of their instruments, and devices with carbolic acid […] after attending a birth, and each use on pregnant women or puerpera, regardless of whether or not they are ill. The midwife shall also wash her hands thoroughly in diluted carbolic acid solution.^46^

Kleinwächter’s draft obliged midwives to carry carbolic acid solution with them to all home visits and to make proper use of the disinfectant in order to avoid the ‘passing of pathogenic matter to their patients’, but also so as to protect themselves against criminal charges.^47^ Without waiting for the Ministry’s response to the draft instruction, the Tyrolean state government issued a decree based on the aforementioned paragraph, legally binding midwives to use carbolic acid in their obstetric routines. The decree took effect in the realm of Tyrol and Vorarlberg on 24 January 1878 and in the following weeks all midwives in the country as well as all regional medical officials received a printed version in their respective languages, German or Italian.^48^

News of the innovative legal development in the westernmost province quickly spread across the Austrian Empire. Based on a note received from Kleinwächter, the *Prager medicinische Wochenschrift* (*Prague Medical Weekly*) reported that Tyrol was the first province to successfully introduce compulsory prophylaxis against ‘this scourge of humanity’.^49^ The article concluded that it was far more desirable and necessary to equip midwives with a bottle of carbolic solution than with smelling salts or strong vinegar.^50^ Kleinwächter’s dissemination tactics, going beyond the official, ministerial routes, showed an immediate effect. The governor of Upper Austria inquired whether he could receive a copy of the negotiation documents, thus facilitating debates on the implementation of a similar order within his region.^51^

Although there was local support on both medical and political levels, one voice predominated. Josef Späth (1823–96), Professor of Obstetrics and Gynaecology in Vienna and, as a member of the Empire’s Supreme Medical Council, one of the leading figures in Austrian medical administration, took an unequivocal stand in favour of the proposed revision to the Midwives’ Instruction. At a meeting of the Vienna Medical Council in April 1878 he argued in favour of Ignaz Semmelweis’ practice of compulsory hand washing.^52^ He believed that many male obstetricians had finally overcome their scepticism, especially following Lister’s success in reducing wound infection.^53^ Nevertheless, puerperal fever was not under control, and Späth recounted several cases from his own private practice where infection had most likely been transmitted by midwives. By shifting attention to the hygiene conditions in home births, which still accounted for the majority of births in the Austrian Empire,^54^ obstetricians entered a new battlefield in the fight against puerperal fever.

Further, during the following meetings of the Vienna Medical Council it became clear that officials did not want to wait for the outcome of a lengthy debate on a new instruction but were pressing for an immediate implementation of disinfection rules similar to the Tyrolean example. Thus, a supplementary decree to the existing 1874 Midwives’ Instruction was issued on 23 October 1878, making disinfection with carbolic acid obligatory across the Empire.^55^ It is worth noting here that the elite physicians, who were relied upon to follow a professional code, took it for granted that midwives could only be persuaded to follow the new rules via legal enforcement. They did not believe in the possibility that midwives would want to assess the information and adopt the new measures of their own accord but had to be compelled to do so. Thus, the process of implementing puerperal prophylaxis further strengthened male superiority in the field of obstetrics. Obstetricians were the ones who created and conveyed antiseptic knowledge, and those who controlled its transfer and proper application.

Although the Ministry of the Interior disseminated the decree to all regional authorities, only the trendsetter Tyrol and the Moravian Margraviate included it in their official gazettes, thus providing visibility and public outreach.^56^ Some of the remaining provincial elites such as the Lower Austrian government saw no need for legally implementing the decree, choosing instead to merely inform midwives via administrative channels.^57^ Yet others actively protested that the decree did not go far enough in inducing midwives to take up the new measures. In January 1879, the *Centralverein deutscher Aerzte in Böhmen* (Central Association of German Physicians in Bohemia) presented a petition to the Ministry of the Interior calling for more concrete action in preventing puerperal fever.^58^ The commission, comprising the leading figures in the Prague obstetric circle, Professors August Breisky, Johann Streng (1817–87) and Ferdinand von Weber-Ebenhof, criticised Austrian statistics for not providing valid data on the extent of puerperal mortality. First, the official post-mortem certificates did not include a separate category naming ‘childbed fever’ as a cause of death. Second, midwives were not obliged to document their attendances in a diary. The Bohemian experts concluded that this would have to change in order to facilitate a proper evaluation of the preventive measures. Based on the findings of Franz Winckel (1837–1911) for the German territories of Saxony and Prussia, they challenged Léon LeFort’s assumption dating to the 1860s that mortality in home deliveries was generally eight times lower than in maternity clinics.^59^ The Bohemian professors argued that preventive measures had successfully reduced puerperal mortality within the clinical environment. The City of Berlin, for instance, showed significantly lower mortality rates during the 1870s than the rural areas of Prussia. They suspected similar findings could also be produced for Bohemia and the rest of the Empire, yet supportive data was not available.^60^

In addition, they demanded educational reform that would especially target trainees. Displaying traditional male assumptions as to the intellectual capacities of women, the professors argued that a brief practical introduction during their studies would not guarantee midwives’ mastery of antiseptic measures. The supplementary decree was also regarded as inadequate, as it did not set out to thoroughly instruct midwives in the new regime, thus resulting in faulty performances. ‘Our everyday experience regarding the application of prophylactic measures and the midwives’ use of carbolic solution shows us that thorough instruction and guidance must be our greatest concern, as our midwives continuously act against the principles of antiseptic prophylaxis.’^61^ The petitioners concluded that not only should practical instruction be intensified but also a special chapter on disinfection and the prevention of puerperal fever must be included in midwifery textbooks. Overall, this called for a fundamental revision of the existing Midwives’ Instruction.^62^

A Bohemian draft version was forwarded to the Ministry of the Interior, which in turn asked for an evaluation through the Supreme Medical Council situated in metropolitan Vienna. Throughout several meetings from July 1879 to the beginning of 1880 the expert commission was occupied with reasoning on several aspects of the draft. Again, it was Josef Späth who used his power to shape opinions within the Council. In collaboration with Gustav Braun (1829–1911), his successor as chief of the second Vienna Maternity Clinic, Späth delivered his obstetric verdict, meticulously reviewing the draft and restructuring several paragraphs. Although the Supreme Medical Council’s final vote supported the revision, progressive ideas were systematically downplayed in favour of a more traditional approach.^63^

The ambitious Bohemian draft was mitigated both in content and style, but still included the main demands of the petition. Disinfection was a prominent theme, including guidelines on the use of antiseptic agents (carbolic acid), the recommendation to avoid cases of sick-nursing, and the obligation for literate midwives to keep a diary in order to facilitate statistical analysis and control. The new instruction was also intended to strengthen the position of physicians, as midwives were firmly reminded of the limits of their professional competence. Concerning its stylistic presentation, the Bohemian draft had been rather bold, refraining from complicated phrasing using graphical and metaphoric language. Most importantly, the instruction was basic in wording, detailed and explanatory in regards to practices and routines, and consisted of only one document. This style, however, was not adopted in the final version, as the Viennese experts favoured a more concise format. Most of the explanatory information was therefore moved to an appendix which included detailed regulations on puerperal prophylaxis, the consultation of the obstetrician, and the keeping of diaries and birth charts. This appendix, in fact, constituted the main innovation in the Revised Midwives’ Instruction.^64^

This episode illustrates the simmering conflicts between the two leading Austrian universities, which were essentially based on academic competition between the two poles of Vienna and Prague. It is moreover clear proof of the extent of centralised medical administration in the Austrian Empire and the interpretational sovereignty of selected figures in metropolitan Vienna. The Revised Midwives’ Instruction was eventually published in the *Reichsgesetzblatt* (National Gazette) on 4 June 1881.^65^ In its legal implementation of antiseptic measures, Austria became a trendsetter in European obstetrics. Although Prussia, for instance, had established a highly regulated midwifery system throughout the nineteenth century, officials were reluctant to adapt to the accelerated speed of innovations in the fields of microbiology, hygiene and public health. A comprehensive instruction for the prevention of childbed fever was only issued in November 1888.^66^ Interestingly enough, the Swedish government distributed a circular on antiseptic measures in midwifery practice on 13 June 1881, just a little over a week after the Austrian instruction was released. Denmark also followed this example in 1881.^67^ Whether this was a mere coincidence or Swedish and Danish officials had observed the Austrian medical system more closely, is yet to be scrutinised.^68^ Further research is required on the signalling effect that Austrian antiseptic legislation had on the rise of puerperal prophylaxis in midwifery schemes across Europe.

## Knowledge Dissemination and Appropriation

Legal regulation and the passing of bills are one thing, but their actual implementation and legal enforcement represent a completely different challenge. The following section analyses the strategies that prominent midwifery teachers in the Austrian Empire chose to convey knowledge of antiseptic regimes to the midwifery profession. The implementation did not only involve dissemination in a top-down mode; as we shall see, midwives took some control over their own professional development following a lack of commitment from the elites. This, and other aspects of the midwives’ adoption of the new regime, becomes clear when we look at the activities of the first midwifery societies established throughout the 1880s.^69^ Finally, this section looks at the caution with which physicians regarded and handled the transfer of knowledge about the new antiseptic regimes outside of the clinical setting, and at the gendered nature of their dissemination strategies.

According to Josef Späth, the 1881 Revised Midwives’ Instruction simply cemented practices long exercised in the leading Austrian maternity clinics and imparted to midwifery students during their courses.^70^ Späth summarised his educational approach thus: ‘The necessity of strict cleanliness is being perpetually sermonized and impressed upon the students’.^71^ However, he did acknowledge that ‘[t]he instructive word alone is not enough. Most importantly, each instructor and everyone involved in the training has to show, daily and on each occasion, by personal example how convinced they are of the necessity of antiseptic prophylaxis’.^72^ Vienna’s leading obstetrician believed that midwifery instructors acting as role models during midwifery courses were the key to success. Yet, Späth rejected all claims for standardised curricula that would abridge academic freedom.^73^ In contrast to Breisky, who called for a normalisation of educational efforts in the form of a standardised midwifery manual^74^—such as Prussia had provided for its midwives since 1815^75^—Josef Späth believed in the academic code of honour. Applied to the field of obstetrics this meant that he believed the heads of midwifery schools should be compelled to comply with prophylactic measures without the enforcement of a standardised curriculum, simply due to their wealth of expertise.^76^

Yet, although Späth claimed to have convinced himself of Semmelweis’ aetiology of puerperal fever during the 1860s, his *Lehrbuch der Geburtshilfe für Hebammen* (Midwifery Manual) first published in 1869 does not support the image Späth tried to create of himself as a veteran in the fight against puerperal fever.^77^ The 1869 edition does not include instructional guidelines on hand washing and disinfection. Only when the use of carbolic acid had become a legally binding prophylactic measure in 1878 did Späth add more than just basic guidelines for cleanliness. However, even the later editions lacked a separate chapter on puerperal prophylaxis and disinfection. Carbolic acid solution was only mentioned in passing and he did not give clear instructions as to the preparation standards of an effective solution. The language he used lacked clear commitment; he recommended rather than urged and thus may have conveyed a sense of arbitrariness.^78^ All in all, Späth’s midwifery manual showed clear shortcomings and can only be considered a half-hearted attempt. Despite the supremacy of the Viennese professor in the legal negotiations surrounding the new midwives’ instruction, it was his colleagues across the Empire who set the standards for antiseptic instruction as evidenced by the inclusion of the subject in midwifery textbooks. In a way, the Empire wrote back.

One leading figure who readily engaged in the compilation of a manual including state-of-the-art prophylaxis was Ludwig Kleinwächter. His *Lehrbuch der Hebammenkunst* (Manual on the Art of Midwifery) was first published in a German edition in Innsbruck in 1879.^79^ Later the manual was translated into Italian, for the use on the Italian midwifery course offered at the University of Innsbruck each year.^80^ Running like a golden thread through the whole manuscript was the urge to implant the seed of antiseptic knowledge in his midwifery students. Kleinwächter dedicated a special chapter to childbed fever, comprising six detailed paragraphs: one explaining the symptoms of childbed fever, another describing the supposed causes, and four paragraphs giving almost pedantic instructions on disinfection.^81^ The textbook was well-received, at least outside of Austria, as a review by the Stuttgart midwifery instructor Hermann Fehling (1847–1925) proves. He stated that it had been high time for a manual that took a stance on antiseptic measures.^82^ In comparison, the 1878 Prussian Midwifery Manual did not propagate the use of carbolic acid solution.^83^ However, Fehling also mentioned the limited reach that midwifery textbooks in general had, as they were not only restricted in relevance to the legal boundaries of the individual states but also to the ideological attitudes of instructors using them.^84^ Whether Kleinwächter’s manual was used anywhere apart from Innsbruck is not known; it is even doubtful if his successor, Friedrich Schauta (1849–1919), used the book after 1881.

In Prague, the 1878 revised second edition of Ferdinand Ritter von Weber-Ebenhof’s midwifery manual did not include a substantial chapter on prophylaxis.^85^ In 1880, however, he presented a concise manual entitled *Das antiseptische Verfahren in der Geburtshilfe* (The Antiseptic Procedure in Obstetrics).^86^ The brochure was published both in a German and a Czech version,^87^ to provide for the two language groups in Bohemia. Over thirty-one pages Weber-Ebenhof elaborated on the antiseptic procedure applicable to midwifery practice, thus presenting the most comprehensive written record in the early phase of implementing the new regime.

In comparison to Kleinwächter’s textbook Weber-Ebenhof’s manual had more longevity, being republished in 1886, albeit only in a Czech version.^88^ In his introduction Weber-Ebenhof explains that although antiseptic measures had been a vital part of practical teaching before, he had ‘postponed the introduction of those principles ushering a new era in his midwifery manual for as long as the facts had not been widely accepted […] This time has now come and this manual on the antiseptic procedure in obstetrics has thus acquired maturity as an appendix to my textbook.’^89^

In contrast to the comprehensive and expensive midwifery manuals published by Späth and Kleinwächter, Weber-Ebenhof’s Bohemian brochure was better suited to reach not only newly educated midwives but also those who had been qualified for some time.^90^ Midwives, or, at least, literate ones, were thus able to inform themselves about the latest requirements.

Overall, however, the question remained of the extent to which it was possible for midwives who were already qualified and practising to meet the new antiseptic requirements. Although local GPs had been receiving regular official requests to supervise and instruct midwives in their area since 1878,^91^ it is not known whether they actually offered training in preparing antiseptic solutions. In her 1888 report, a Styrian midwife claimed that none of the local physicians had ever shown any interest in the supervision of midwives, neither questioning their approaches nor evaluating the cleanliness of their instruments.^92^ The fact that midwives’ questions, such as those quoted in the introduction to this article, were directed to midwifery newspapers also suggests that local physicians were either not trusted as adequate sources of knowledge by practising midwives or simply lacked experience in handling antiseptics themselves. Furthermore, although the Vienna Medical Council had called for the establishment of so-called ‘refresher courses’ for practising midwives, by 1888 only the Carinthian government had invested in such an endeavour.^93^ Subsequently, the topic entered the discussions of the Supreme Medical Council in 1890,^94^ and the intention to mandate refresher courses was included in the 1897 Midwives’ Instruction.^95^ However, leading obstetricians were still criticising the lack of targeted action in this area at the turn of the century.^96^

As a true commitment towards increasing the quality of midwifery education was not forthcoming from the Austrian government, dedicated midwives took direct control of their professional development. The establishment of midwifery societies, first emerging in Vienna in the early 1880s, has to be seen as an immediate reaction to the implementation of antiseptic measures. In these societies midwives actively allied with physicians, inviting them to give lectures on advances in the field of obstetrics. These medical insights were published in the societies’ newspapers, addressing midwives across the Austrian Empire.^97^

Examination of the *Midwifery Newspaper* (*Hebammen-Zeitung*), first launched in October 1887, shows that the topic of antisepsis was repeatedly discussed not only in guest lectures but also in numerous printed letters to the editors, both illustrating the midwives’ conviction as to the importance of the new measures and elucidating the obstacles that still had to be overcome. Besides poor earnings, which often rendered the purchase of sufficient supplies impossible, the resistance of some mothers to the measures as well as the scheming of some, especially older, midwives, were criticised. A midwife from Styria accused her older colleagues of trying ‘to convince the women that this humbug, as they call antisepsis […], is not necessary at all. On the contrary, it would only cause the mothers further pain, due to intense smarting.’ She concluded that awareness should not only be raised among midwives but also among the public.^98^

This was also a claim held by obstetricians. In his manual, Weber-Ebenhof intended to address not only midwives, but to serve as an ‘instruction for all women’.^99^ Demanding that women should know what to require from a good midwife, the instructor claimed that women must not be afraid to inform themselves. Weber-Ebenhof propagated direct patient responsibility to spare women the fate ‘that as yet is wreaking havoc among the ranks of happy spouses and mothers.’ Antiseptic measures, he believed, should become the ‘intellectual property of all women, to whom life is dear and valuable.’^100^ He envisioned a public convinced by the factual accuracy of the aetiology of puerperal fever, diligently supplying themselves with carbolic acid solution in preparation for a delivery. In this bourgeois ideal, knowledge protected mothers from ignorant midwives and gave them agency and power. Thus, he continued, ‘a midwife who neglects the disinfection, will have to bear the humiliation of being urged to thoroughly cleanse her hands by her patient’.^101^ The notion of lay intervention into hygiene standards was not new, as even before the topic of disinfection became legally binding, a resourceful Vienna inner-city pharmacist used one of the leading Austrian newspapers (*Neue Freie Presse*) to inform the public about the need to disinfect. Families expecting a baby, the pharmacist proclaimed, should observe strict hygienic standards and should therefore keep ‘Berger’s Carbolic Soap’ in stock. As it was doubted that midwives, doctors or nurses would always carry disinfectant solutions with them, families were advised to equip themselves for their own protection.^102^ Although the advertised soap probably did not meet the medical standards of antisepsis, the resourceful entrepreneur succeeded in drawing broader attention to a widely discussed medical issue of the time.

The Austrian experts charged with disseminating the new regime to the midwifery profession were obviously also preoccupied with the extent of the publicity they wanted to create. In a time of constant flux and innovation, yet with unresolved matters of aetiology, some experts exercised a certain reluctance to put their ideas down on paper and expose themselves to critics—perhaps mindful of the career trajectories of contemporaries such as Semmelweis or Mayrhofer.^103^ It was one thing to act in accordance with antiseptic rules within the restricted and secure space of one’s own clinic, but yet another to openly propagate a certain procedure. Although later generations, and especially his disciples, saw Josef Späth as a pioneer in the Austrian antiseptic movement, his reservations are visible in his work. Weber-Ebenhof’s introductory remarks to his manual also support the notion of a certain caution surrounding the transfer of antiseptic knowledge beyond the clinic.

In their attempts to raise awareness of the new regime, male experts found clear words, yet often resorted to stereotypical reasoning. They unanimously blamed the ignorance of midwives for maternal deaths. ‘Neither location nor status saves the poor mother if she is in the hands of a careless, unscrupulous midwife. The best care, the largest fortune does not prevent the rich succumbing to childbed fever as do the poor’,^104^ Kleinwächter, for instance, argued. While Späth thought it important that midwives were aware of the dangers that lay in negligence,^105^ Kleinwächter referred to antisepsis as a ‘holy duty’.^106^ In the same tone, the Prague midwifery instructor Weber-Ebenhof proclaimed it the midwife’s duty to bring the beneficent advances of science to fruition in each and every case. He even recommended that those sceptical of the aetiology of childbed fever and ambivalent about puerperal prophylaxis had better abandon their practice.^107^ Besides making accusations of carelessness, Austria’s leading midwifery instructors generally doubted that midwives could comprehend the full extent of the antiseptic innovation.^108^ The poor intellectual capacities of women were one line of argument, lack of education yet another, as the Bohemian obstetrician Emil Ekstein stated in the course of intensified reform efforts.^109^

## Routines and Materials

In many ways, disinfection did not revolutionise midwifery practice but complemented traditional attitudes towards cleanliness. The demands that midwives should dress immaculately, keep their hands clean, nails short, and skin smooth were far from new. That midwives should refrain from hard agricultural work or other labour was a recurring demand made by the majority of midwifery textbooks. What was, in fact, changing throughout the 1870s and 1880s was the definition of cleanliness. The following section thus examines the way in which scientific perceptions changed and affected everyday routines in midwifery practice.

Ever since the onset of pre-bacteriological thinking, introduced into obstetrics by agents such as Ignaz Semmelweis, James Young Simpson or Joseph Lister, it was recognised that washing with soap and water was not enough. Cleanliness was no longer measured by what was visible, but extended far beyond, as the ‘malicious poison’ causing childbed fever could not be seen with the naked eye. Kleinwächter suggested a multisensory approach, including the sense of smell. ‘The best means to discern whether the finger still carries the malicious poison is the sense of smell. As long as it reeks, it is a sign that it is still befouled and that one is still able to infect a healthy woman in childbed.’^110^ Kleinwächter warned midwives that this fetid smell, together with the putrid matter, would not vanish quickly, as blood, pus and vaginal discharge could not be easily washed away with soap and water. In the new antiseptic mind-set, washing with disinfectant solutions was the only appropriate measure of prophylaxis. Weber-Ebenhof concluded that only by the strictest compliance ‘will the tiny enemies of mankind that can only be unmasked with the help of a microscope, be disarmed and many a tear be spared’.^111^

Ludwig Kleinwächter suggested a detailed step-by-step instruction for a procedure midwives could easily follow:

After the hands have been thoroughly washed using soap and nailbrush, carbolic solution is poured onto the hands and the fingers are given a proper rub with the brush. […] Every midwife must have shiny, pure nails without dirty rims. After washing, the nails must be dried and cleaned with great care. Before internal examinations, dip the finger into carbolic oil and ensure that also the nail is properly greased. If the midwife adheres to these precautions, she will not be able to communicate childbed fever from one woman to another. As a result, her reputation among the population will increase.^112^

In this context, Weber-Ebenhof suggested that midwives should always disinfect their hands and forearms in front of their patients, so as to enhance public awareness and appreciation of antisepsis.^113^

Besides taking care of their own hand disinfection, midwives were compelled to prepare a clean birthing environment and to disinfect the parturient and lying-in women. The report of a Viennese midwife shows how challenging this could actually be. The case of a first-time mother who had given birth under dire conditions, lacking the most basic supplies such as clean linen, almost resulted in the death of the young woman. Against all recommendations, her family had left her lying on the blood- and amniotic-fluid-soaked mattress for several days until she developed a fever.^114^ Another midwife from Vienna complained that she was the victim of gossip, as one of the women she had attended had died. However, the woman had already been running a temperature during labour. Due to her oath, she could not have rejected a parturient woman who was ill, the midwife argued.^115^ The recommendation to use only one hand to touch the mother and child while attending a birth, so as to keep the other clean in case one was called to another home delivery shortly after, was hardly feasible.^116^ Therefore, the *Midwifery Newspaper* suggested that due to the growing importance of puerperal prophylaxis, especially sought-after midwives should refrain from taking over such critical cases and leave them to younger colleagues who did not risk losing their established clientele.^117^

Preparatory routines in active birth attendance changed. In order to avoid self-infection, pregnant women were reminded to bathe more frequently than usual. The fear of self-infection was also the reason why Weber-Ebenhof recommended vaginal irrigation when labour started. Midwives should use a weak two per cent solution of carbolic acid for internal disinfection. It is noteworthy that Späth and Kleinwächter did not support this procedure in their textbooks. Kleinwächter later openly criticised internal manipulations on healthy women as an excessive and harmful approach.^118^ Special attention was given to the parturient’s thighs and hands, which had to be disinfected as well.^119^ In any case, the male experts insisted, washing should always be administered with the utmost care, never exposing the naked female body. The advice of acting under covers should not only prevent women from chilling but also guarantee moral demeanour.^120^ Some materials such as sponges were banned from the birthing room, as they were especially hard to clean and could hold large proportions of infectious matter in their cavities. Instead, washed and carbolised pieces of linen were used in a disposable system, being burnt after use.^121^ Cotton wool (*Bruns’sche Watte*) was seen as the most convenient fabric to absorb lochia after birth.^122^

Ideally, antiseptic measures were not restricted to the immediate attendance in home deliveries but reached far beyond. Midwives had to integrate washing with carbolic acid solution into their daily routines. After each delivery clothes had to be changed and midwives were reminded to take soap baths in tepid water.^123^ The midwife was not only seen as a medical professional but also exercised a social hygienist function, as she had access to the homes of all classes. Besides recommending general improvements in hygiene, for example concerning the position of dung heaps or privies, she had to actively disinfect the latter by pouring in carbolic acid solution or applying carbolic powder.^124^ Strict adherence to the scheme would not only prevent childbed fever from spreading in communities but could also be a lifesaver for midwives themselves, as it prevented infection with diseases such as syphilis or smallpox.^125^

Carbolic acid was regarded the most effective disinfectant at the time, yet the preparation of a non-toxic solution was tricky. Midwives had to follow the dilution guidelines carefully in order to get the mixing proportions right. Their stocks had to be locked away in a cool, dry place, out of reach of unauthorised persons. They were expected to prepare a saturated solution that could be taken along to home deliveries. ‘From this concentrated carbolic acid solution the midwife has to make the desired carbolic water in the dwelling of the pregnant, parturient or confined woman.’^126^ For this purpose a new device entered the midwives’ bag: a cylindrical glass container with a capacity of 30 grams and a trinomial scale, indicating the percentage (1–3 per cent) of the dilution. To produce a one per cent carbolic acid solution, midwives were instructed to pour the saturated liquid into the container up to the first scale mark. Then the saturated solution should be poured into one litre of pure, boiled but cooled off water.^127^ Both Kleinwächter and Weber-Ebenhof included a picture of the glass container in their guidelines.^128^

What appeared to be a simple and easy to implement measure in the clinical environment could present the midwives with unexpected problems during home deliveries. A midwife from Traismauer in Lower Austria related that the main problem she faced was the availability of clean water. In order to clear the water from obvious dirt such as wood and plant particles she recommended a filter made of an enamel funnel and cotton wool. The freshly boiled filtered water could best be collected in the large wine bottles available in every household in her region, the midwife added.^129^

Despite its effectiveness, August Breisky repeatedly and openly doubted the applicability of carbolic acid in midwifery practice, as he did not believe women capable of mastering the complex chemical process. He reported on several cases of corrosive injuries on the female genitals and surrounding skin tissue, which had come about due to faulty preparation. ‘In one case, the puerpera even suffered life-threatening erysipelas originating from a chemical burn’.^130^ The Prague circle preferred disinfection with the less-effective potassium permanganate, as it was non-toxic and easier to prepare. The dark red crystals quickly dissolved in clear water, producing a pinkish solution. After some time, this pink solution would turn brown, signalling the loss of its potential capacities. The leading obstetricians at Prague University found this colour code especially compelling, as midwives would easily be able to discern from the colour whether a solution was still useable.^131^

Aware of these debates, Weber-Ebenhof included a list of several other antiseptic agents applicable in midwifery practice. Yet, salicylic acid, besides being hard to dissolve, was very expensive; and chlorinated lime, denatured alcohol, strong spirit with soap, strong vinegar, and chlorine water all showed less reliable effects.^132^ However, the experts involved were well aware that they lived in a time of constant flux, acknowledging that what then was seen as the most reliable chemical could soon be replaced by something more effective.^133^

As a matter of fact, carbolic acid remained the most popular antiseptic agent used in midwifery practice until well after the turn of the century. The highly effective and less expensive Sublimat, a mercury compound invented in the early 1880s, remained taboo for midwives. Because of the danger of fatal poisoning, only doctors were allowed to use mercuric chloride. The positive side-effects of the colour- and odourless liquid, namely its skin-friendly effect, were thus reserved for male obstetricians and their patients.^134^ Meanwhile, the repeated requests to the *Midwifery Newspaper* regarding hand care products give an insight into the consequences of the implementation of carbolic acid on midwives’ skin.^135^

A topic controversially discussed by midwives and male obstetricians was cost effectiveness. Midwives did not receive any compensation for the costs of disinfectant solutions.^136^ For many rural midwives, who often attended only a few births per annum, this meant an enormous expense.^137^ While some obstetricians naively argued that the community benefit outweighed the cost argument and midwives should not take the loss of earnings ‘so much to heart’,^138^ midwifery teachers in particular took a critical view of the situation. Together with the midwifery societies they demanded that the state take over the additional costs.^139^ In 1900, Ludwig Piskaček (1854–1932), the head of the Linz Midwifery Institute, calculated a rise in the costs of home deliveries by almost one third due to the cost of materials. Piskaček assumed that the majority of midwives in Upper Austria only administered ‘pseudo-antiseptic’ measures, using highly diluted and thus ineffective solutions. As parturient women and their families were not willing to accept extra costs due to the application of antiseptic measures, midwives had to keep their expenses manageable. The only way to ensure the application of antiseptic standards in home deliveries, in his opinion, was to provide midwives with regular incomes above the level of precarity.^140^ However, these demands went unheeded; the reform of the salary system was frequently postponed and not realised until well after the collapse of the Austrian Empire.^141^

## Conclusion

Ever since Ignaz Semmelweis presented his scientific findings in 1847, efforts in puerperal prophylaxis had caused highly ambivalent reactions in Austrian obstetrics. While his aetiology was controversial and widely debated, knowledge of the effectiveness of chlorine washing seems to have persisted. As a result, Lister’s system of wound treatment was not completely new to obstetricians in the mid-1860s and the antiseptic method using carbolic acid was quickly integrated into their routines. By the end of the 1870s the leading figures in Austrian obstetrics—especially the heads of the large maternity units in Vienna and Prague—unanimously claimed to have successfully adopted antiseptic measures in their clinics. As soon as puerperal mortality was declining within the clinical context, the physicians in charge tried to translate and transfer their practices to home deliveries across the Austrian Empire. Midwifery education received increased attention and, besides disseminating practical knowledge during training courses, a revision of midwifery legislation was prompted. Over the course of five years, efforts led to several decrees, culminating in the issuing of the 1881 Revised Midwives’ Instruction. The Austrian Empire was indeed at the forefront of puerperal prophylaxis, being the first to pass far-reaching legal regulations requiring midwives to use carbolic acid.

In the case of both midwifery and obstetrics, the antiseptic transformation has to be read as a generational issue. Younger proponents such as the Innsbruck professor Ludwig Kleinwächter forged ahead, championing and inducing fundamental change, while eminent authorities in the field of obstetrics showed more reluctance to take an open position. Whether this is to be understood as an ideological reaction of a new generation of obstetricians to the evident reluctance of the past, or as emerging simply out of career considerations, remains speculative. What is visible, though, is the increasing appreciation of evidence-based research and the accelerated speed with which new approaches were put to the test. What can also be detected is the pressure of competition between the leading clinical institutions in the Austrian Empire. Despite the strong commitment towards antisepsis displayed at universities in the various crown lands of the Empire (i.e. Bohemia/Tyrol), the interpretational sovereignty and legislative power remained firmly anchored in Vienna, the Empire’s intellectual centre.

Expert opinions may have differed on the practicability of carbolic disinfection and the means of knowledge dissemination but, beyond the clinical context, obstetricians stood united: midwives had to be trained and supervised by male experts. From a gendered perspective, it was not enough to provide midwives with all the information on antiseptic measures and rely on their adopting the routines as a matter of common sense; rather, their compliance had to be legally enforced. As nineteenth-century male obstetricians strongly believed in the reduced intellectual capacities of women, midwives could be trusted neither to grasp the significance of their actions, nor to adhere to the code of conduct as a matter of professional conscience. Besides being one of the most influential laws in the history of midwifery and public health, the 1881 Revised Midwives’ Instruction also stands as evidence of the paternalistic nature of Austrian obstetrics. The legal regulation further entrenched the subordinate position of midwives in the medical hierarchy, legitimising the increased control of the male medical elite.

The introduction of antiseptic birth attendance routines also changed the practice of midwifery. Midwives had to integrate new techniques into their procedures, and familiarise themselves with new materials and instruments. Most importantly, they had to raise public awareness of the benefits of antisepsis, bringing the notion of disinfection into private birthing rooms. However, those willing to take on board the scientific advances and eager about practising antiseptic measures faced various obstacles. Besides intra-professional frictions and resistance from parturient women, economic reasons continually impeded proper implementation. As midwives were compelled to purchase antiseptic agents at their own expense, many could simply not afford to support the advances. Although the numerous reports quoted in the Austrian *Midwifery Newspaper* perfectly illustrate the recurring struggles and the unwillingness of administrative and political institutions to take remedial actions, it is still not clear whether and how violation of the law was actually punished. This calls for a more thorough investigation of both the midwives’ and patients’ perspective on the most influential innovation in midwifery practice throughout the nineteenth century. Medical visitation protocols as well as criminal court records could hold evidence of the extent of the control exerted over midwives and the public.

## Footnotes

1 B.B., ‘Letter to the editors’, *Hebammen-Zeitung. Organ des Unterstützungs-Vereines für Hebammen*, 1887, 3, 7.

2 M.M., ‘Letter to the editors’, *Hebammen-Zeitung*, 1887, 4, 6.

3 M.J., ‘Letter to the editors’, *Hebammen-Zeitung*, 1887, 5, 6.

4 Michael Worboys, *Spreading Germs. Disease Theories and Medical Practice in Britain, 1865–1900* (Cambridge: Cambridge University Press, 2000); K. Codell Carter, *The Rise of Causal Concepts: Case Histories* (Abingdon: Ashgate Publishing, 2003); in the obstetrical context see also Sydney Selwyn, ‘Sir James Young Simpson and Hospital Cross-Infection’, *Medical History*, 1965, 9, 241–8; Constance E. Putnam, ‘Simpson and Semmelweis: Debating the Contagiousness of Puerperal Fever’, in Alison Nuttall, Rosemary Mander, eds, *James Young Simpson: Lad o Pairts* (Erskine: Scottish History Press, 2011), 76–94.

5 Michael Worboys, ‘The History of Surgical Wound Infection: Revolution or Evolution?’, in Thomas Schlich, ed, *The Palgrave Handbook of the History of Surgery* (London: Palgrave Macmillan, 2018), 215–33; Thomas Schlich, ‘Asepsis and Bacteriology: A Realignment of Surgery and Laboratory Science’, *Medical History*, 2012, 56, 308–334; Thomas Schlich, ‘”The Days of Brilliancy are Past”: Skill, Styles and the Changing Rules of Surgical Performance, ca. 1820–1920’, *Medical History*, 2015, 59, 379–403; Johanna Bleker ‘Antisepsis in Deutschland. Zur Wissenschaftsgeschichte der Chirurgie 1872–1892’, *Medizinhistorisches Journal*, 2013, 48, 273–305; Peter J. Kernahan, ‘Causation and Cleanliness: George Callendar, Wounds and the debates over Listerism’, *Journal of the History of Medicine and Allied Sciences*, 2009, 64, 1–37; for a more specific approach to antisepsis and asepsis in the Austrian Empire see Heinz Flamm, ‘Von der Antiseptik zur Aseptik’, *Wiener Klinische Wochenschrift*, 2007, 119, 685–96.

6 A similar approach was chosen by Anne Hanley, ‘“Scientific Truth into Homely Language”: The Training and Practice of Midwives in Ophthalmia Neonatorum, 1895–1914’, *Social History of Medicine*, 2013, 27, 199–220; Claire L. Jones *et al.*, ‘Personalities, Preferences and Practicalities: Educating Nurses in Wound Sepsis in the British Hospital, 1870–1920’, *Social History of Medicine*, 2017, 31, 577–604.

7 Birgit Bolognese-Leuchtenmüller*, Bevölkerungsentwicklung und Berufsstruktur, Gesundheits- und Fürsorgewesen in Österreich 1750–1918* (Wien: Verlag für Geschichte und Politik, 1978), 155–61 and 252. On the problematic composition of Austrian medical statistics also see: Elisabeth Dietrich-Daum, ‘Reporting Death. The Case of Austrian Tuberculosis Mortality Registration—Critique and Consequences for Historical Epidemiology’, *Prague Medical Report*, 2009, 110, 146–58.

8 Worboys, *Spreading Germs*, 99–107.

9 The term ‘Austrian Empire’ is used to describe the Cisleithanian part of the Dual Monarchy (Austria-Hungary). Geographically it comprised all royal domains, the so called “crown lands” from Vorarlberg and Tyrol in the west to Bukovina in the east and from Bohemia in the north to Dalmatia in the south. The Hungarian territories are excluded due to their independent legislative development since 1867.

10 Sonia Horn, ‘A Model for All? Healthcare and the State in 18^th^ Century Habsburg Inherited Countries’, in Laurinda Abreu, Patrice Bordelais, eds, *The Price of Life: Welfare Systems, Social Nets and Economic Growth* (Lisbon: PhoenixTN, 2008), 303–15; Marina Hilber, ‘Professionalisierung wider Willen? Die Ausbildung von Hebammen in Tirol und Vorarlberg im Spannungsfeld von Norm und Aushandlung’ *Geschichte und Region, storia e regione*, 2015, 24, 73–96.

11 Friedrich Schauta, ‘Die österreichischen Gebäranstalten in den Jahren 1848 bis 1898’, in Hans Heger, ed, *Oesterreichs Wohlfahrts-Einrichtungen 1848–1898. Festschrift zu Ehren des 50jährigen Regierungs-Jubiläums seiner k.u.k. Apostolischen Majestät des Kaisers Franz Joseph I.*, vol. 3: Gesundheitspflege (Wien: Moritz Perles, 1900), 264–83, 271.

12 Sigrun Bohle, *Hebammen. Zur Situation der Geburtshelferinnen im endenden 19. und beginnenden 20. Jahrhundert in Österreich* (unpublished MA thesis, Vienna University, 1985).

13 Ludwig Piskaček, ‘Zur Reform des Hebammenwesens in Oberösterreich’, *Das österreichische Sanitätswesen*, 1900, 41–4, 461–5, 473–6, 485–9, 498–502; Emil Ekstein, ‘Zur Sozialhygiene des Puerperalfiebers’, *Gynäkologische Rundschau*, 1907, 3–4, 124–6, 161–5.

14 Bolognese-Leuchtenmüller*, Bevölkerungsentwicklung*, 276.

15 Adalbert Zaleisky, *Handbuch der Gesetze und Verordnungen welche für die Polizei-Verwaltung im österreichischen Kaiserstaate von 1740–1825 erschienen sind*, vol 2 (Wien: Friedrich Manz, 1854), 33.

16 ‘Instruction für Hebammen’, *Reichsgesetzblatt für die im Reichsrathe vertretenen Königreiche und Länder*, 1874, 32, 31–3.

17 Ulf Högberg, *Maternal Mortality in Sweden* (Umeå: Umeå University, 1985); Ulf Högberg, ‘The Decline of Maternal Mortality in Sweden’, *American Journal of Public Health*, 2004, 94, 1312–20.

18 Irvine Loudon, *Death in Childbirth: An International Study of Maternal Care and Maternal Mortality 1800*–*1950* (Oxford: Clarendon Press, 1992).

19 Irvine Loudon, ‘Midwives and the Quality of Maternal Care’, in Hilary Marland, Anne Marie Rafferty, eds, *Midwives, Society and Childbirth. Debates and Controversies in the Modern Period* (London/New York: Routledge, ^2^1999), 180–200.

20 Anne Løkke, ‘The “Antiseptic Transformation” of Danish Midwives, 1860–1920’, in Hilary Marland, Anne Marie Rafferty, eds, *Midwives, Society and Childbirth. Debates and Controversies in the Modern Period* (London/New York: Routledge, 1999), 102–33.

21 Jürgen Schlumbohm, ‘Did the Medicalisation of Childbirth reduce Maternal Mortality in the Eighteenth and Nineteenth Centuries? Debates and Data from Several European Countries’, in William H. Hubbard *et al.*, eds, *Historical Studies in Mortality Decline* (Oslo: Novus Forlag, 2002), 96–112; Vincent De Brouwere, ‘The Comparative Study of Maternal Mortality over Time: The Role of the Professionalisation of Childbirth’, *Social History of Medicine*, 2007, 20, 541–62; Robert Woods, ‘Medical and Demographic History: Inseparable?’, *Social History of Medicine*, 2007, 30, 483–503.

22 Stephan Curtis, ‘Midwives and their Role in the Reduction of Direct Obstetric Deaths during the late Nineteenth Century: The Sundsvall Region of Sweden (1860–1890)’, *Medical History*, 2005, 49, 321–50. For birth-related patient agency in Austria see Marina Hilber, ‘”[…] aus freyer Wahl und Zutrauen […]”: Eine patientinnen-orientierte Fallstudie zum Wahlverhalten von Gebärenden im inneralpinen Raum Tirols und Vorarlbergs um 1830’, *Medizin, Gesellschaft und Geschichte. Jahrbuch des Instituts für Geschichte der Medizin der Robert Bosch Stiftung*, 2018, 36, 11–41.

23 Loudon, Death in Childbirth, 49–84; Schlumbohm, ‘Medicalisation of Childbirth’, 96–112.

24 Ludwig Kleinwächter, *Lehrbuch der Hebammenkunst* (Innsbruck: Verlag der Wagner’schen Universitätsbuchhandlung, 1879), 286–7. Otto von Herff, ‘Das Kindbettfieber’, in Franz von Winckel, ed, *Handbuch der Geburtshülfe*, vol. 3, part 2 (Wiesbaden: Verlag von J.F. Bergmann, 1906), 332–461, 383.

25 K. Codell Carter, ‘Ignaz Semmelweis, Carl Mayrhofer, and the Rise of Germ Theory’, *Medical History*, 1985, 29, 33–53, 44; Loudon, *Death in Childbirth*, 77–9.

26 Ludwig Kleinwächter, *Grundriss der Geburtshülfe für Praktische Aerzte und Studirende* (Wien: Urban & Schwarzenberg, 1877), 315–6; Loudon, *Death in Childbirth*, 57–68.

27 In Prague, for instance, the washings had been abandoned as ineffective after only short-term trials. Friedrich Wilhelm Scanzoni, ‘Josef Škoda’s Ueber die von Dr. Semmelweis entdeckte, wahre Ursache der in der Wiener Gebäranstalt ungewöhnlich häufig vorkommenden Erkrankungen der Wöchnerinnen [review]’ *Ergänzungsblätter zur Vierteljahrschrift für die Praktische Heilkunde*, 1850, 7, 25–33.

28 Emil Ehrendorfer, ‘Ueber antiseptische (locale) Behandlung in der Geburtshülfe’, *Archiv für Gynäkologie*, 1886, 27, 171–226; Julius Pagel/Karl Sudhoff, *Einführung in die Geschichte der Medizin in 25 akademischen Vorlesungen* (Berlin: S. Karger, 1915), 520–3. Worboys, ‘History of Surgical Wound Infection’, 219–20.

29 August Breisky, ‘Unsere Stellung zur neuen Gebäranstalt. Antrittsvorlesung bei Eröffnung der II. Geb.-Klinik für Aerzte an der neuen Gebäranstalt zu Prag am 26. April 1875’, *Aerztliches Correspondenzblatt: Organ des Vereines deutscher Aerzte in Prag*, 1875, 27, 170–6.

30 Ursula Zimmermann, ‘August Breisky: Sein Leben und Wirken als Arzt und Lehrer’ (unpublished PhD thesis, Vienna University, 1941), 95–100.

31 Jan Dvořák, ‘Nástin zajímavých událostí od 1 května 1878 do konce dubna 1879’, *Časopis Lékařův českých*, 1879, 26–36, 201–2, 209–11, 225–8, 258–60, 281–4.

32 Ehrendorfer, ‘Behandlung’, 179–94.

33 Österreichisches Staatsarchiv (Austrian National Archive, referred to as ÖStA in the following), Allgemeines Verwaltungsarchiv (AVA), Ministerium des Inneren, Allgemein 36/2, Karton 951, 1877, Zl. 9950, 7683.

34 Jan Surman, ‘Vom “akademischen Altersheim” zur Spitzenforschungsanstalt? Mobilität der Wiener Professoren 1848–1918’, in Mitchell G. Ash/Josef Ehmer, eds, *Universität—Politik—Gesellschaft* 2 (Vienna: V & R unipress/Vienna University Press, 2015), 621–48.

35 Felicitas Seebacher, *Das Fremde im „deutschen“ Tempel der Wissenschaften. Brüche in der Wissenschaftskultur der Medizinischen Fakultät der Universität Wien* (Wien: Verlag der Österreichischen Akademie der Wissenschaften, 2011), 131–2; Felicitas Seebacher, ‘Carl Freiherr von Rokitansky und Theodor Billroth. Politisierte Wissenschaftsstile an der Medizinischen Fakultät’, in Ash/Ehmer, eds, *Universität—Politik—Gesellschaft*, 197–208.

36 ÖStA, AVA, Ministerium des Inneren, Allgemein 36/2, Karton 951, 1877, Zl. 9950, 7683.

37 Besides the medical faculties at the Universities of Vienna, Prague, Graz and Innsbruck, where surgeons, physicians and midwives were trained alongside each other, institutions dedicated solely to educating midwives were established throughout the Austrian Empire, usually in the provincial capitals (Krakow, Brno, Linz, Lviv, Klagenfurt, Ljubljana, Salzburg, Olomouc, Trieste, Czernowitz and Zara). Schauta, ‘Gebäranstalten’, 264–83.

38 See Bleker’s claim that the history of surgical antisepsis is devoid of ‘heroic figures’ and supports itself through the collective effort of mostly unnamed advocates, predominantly assistants and low-level personnel. Bleker, ‘Antisepsis in Deutschland’, 296–8.

39 Karl Mayrhofer, ‘Über das Vorkommen von Vibrionen bei Wöchnerinnen’, *Wochenblatt der Gesellschaft der Ärzte*, 1863, 19, 17–9; Karl Mayrhofer, ‘Untersuchungen über Ätiologie der Puerperalprocesse’, *Medizinisches Jahrbuch*, 1863, 19, 28–42; Karl Mayrhofer, ‘Zur Frage der Ätiologie der Puerperalprozesse’, *Monatsschrift für Geburtshilfe*, 1865, 25, 112–4; after being rejected in the application process, Mayrhofer left Austria for Russia and tried to establish a medical practice first in Tbilisi, later in Saint Petersburg. Whether his morphine addiction led to professional failure or vice versa is not clear. He died in 1882. For a critical interpretation of Mayrhofer’s role in germ theory see: Carter, ‘Ignaz Semmelweis’, 37–53.

40 Karl Rokitansky, ‘Untersuchungen der mikroskopischen Zusammensetzung der Lochien’, *Medizinisches Jahrbuch*, 1874, 30, 161–78.

41 Erna Lesky, *Die Wiener Medizinische Schule im 19. Jahrhundert* (Graz/Köln: Verlag Hermann Böhlaus Nachfolger, 1965), 467–78.

42 Joseph Amann, *Klinik der Wochenbettkrankheiten* (Stuttgart: Verlag von Ferdinand Enke, 1876).

43 Kleinwächter, *Grundriss*, VI. In 1876 a Czech obstetric manual was published, which also laid a clear emphasis on antiseptic procedures. Kleinwächter knew this manual and honourably mentioned its focus on prophylaxis, however, Křižek's manual had little impact as it addressed only a limited readership among Czech speaking Bohemian doctors. Čeněk Křižek, *Základové Porodnictví pro Lékaře. Se zvláštním zřetelem k části operativní* (Praze: Tiskem a nákladem Knihtiskárny Josefa Koláře, 1876).

44 ÖStA, AVA, Ministerium für Cultus und Unterricht, Personalakte Kleinwächter.

45 Antisepsis was not new at the Innsbruck clinics, as the surgeon Eduard Albert (1841–1900) had introduced antiseptic measures to his wards as early as 1873. Flamm, ‘Antiseptik zur Aseptik’, 690.

46 Tiroler Landesarchiv (Tyrolean State Archive, referred to as TLA in the following), Statthalterei für Tirol und Vorarlberg, Sanität 1878, Karton 2116, Zl. 1303.

47 TLA, Statthalterei für Tirol und Vorarlberg, Sanität 1878, Karton 2116, Zl. 1303. Worboys mentions a British court case, where midwives were prosecuted for ‘manslaughter by infection’. Worboys, *Spreading Germs*, 104.

48 TLA, Statthalterei für Tirol und Vorarlberg, Sanität 1878, Karton 2116, Zl. 1303.

49
*Prager medicinische Wochenschrift* 1878, 9, 89–90.

50
*Ibid*.

51 TLA, Statthalterei für Tirol und Vorarlberg, Sanität 1878, Karton 2116, Zl. 1303.

52 The literature on Ignaz Semmelweis and his tragic career is extensive. See for example: Erna Lesky, *Ignaz Semmelweis und die Wiener Medizinische Schule* (Graz/Wien/Köln: Hermann Böhlaus Nachfolger, 1964); K. Codell Carter and Barbara R. Carter, *Childbed Fever: A* *Scientific* *Biography of Ignaz Semmelweis* (Westport-London: Greenwood Press, 1995).

53 Späth [or Spaeth] had publicly acknowledged the usefulness of washing as early as 1864, however, the extent of its application in the Vienna Maternity Clinic throughout the 1860s is not clear. Josef Spaeth, ‘Über die Sanitätsverhältnisse der Wöchnerinnen an der Gebäranstalt für Hebammen in Wien’, *Medizinisches Jahrbuch der k.k. Gesellschaft der Ärzte in Wien*, 1863, 19, 10–27; Josef Spaeth, ‘Statistische und historische Rückblicke auf die Vorkommnisse des Wiener Gebärhauses während der letzten 30 Jahre’, *Medizinisches Jahrbuch der k.k. Gesellschaft der Ärzte in Wien*, 1864, 20, 145–64. Lesky, *Wiener Medizinische Schule*, 218–9. Carter , Carter, *Childbed Fever*, 70; Worboys, ‘History of Surgical Wound Infection’, 220–3.

54 Up until 1900, 95–8 per cent of all women in Bohemia gave birth at home. Teresa J. Balkenende, *Protecting the National Inheritance: Nation-State Formation and the Transformation of Birth Culture in the Czech Lands, 1880–1938* (unpublished PhD thesis: University of Washington, 2004), 3.

55 ÖStA, AVA, Ministerium des Inneren, Allgemein 36/2, Karton 951, 1878, Zl. 10381.

56 ‘Verordnung des k.k. Statthalters vom 20. November 1878, betreffend den Gebrauch der Carbolsäure von Seite der Hebammen bei Ausübung ihres Berufes’, *Gesetz- und Verordnungsblatt für die gefürstete Grafschaft Tirol und das Land Vorarlberg*, 1878, 53, 57. ‘Kundmachung des k.k. Statthalters in Mähren vom 28. October 1878, betreffend eine Nachtrags-Bestimmung zur Instruction für Hebammen’, *Landes-Gesetz- und Verordnungsblatt für die Markgrafschaft Mähren*, 1878, 24, 119.

57 ÖstA, AVA, Ministerium des Inneren, Allgemein 36/2, Karton 951, 1879, Zl. 1048.

58 ÖStA, AVA, Ministerium des Inneren, Allgemein 36/2, Karton 951, 1879, Zl. 605. August Breisky, ‘Petition des Centralvereins deutscher Aerzte in Böhmen “zur Verhütung des Puerperalfiebers nöthigen Reformen im Unterrichte und in den Instructionen der Hebammen“’, *Prager medicinische Wochenschrift*, 1879, 3, 27–9.

59 Ibid.; Schlumbohm, ‘Medicalisation of Childbirth’, 109; Léon LeFort, *Des Maternités: Etude sur les Maternités et les institutions charitables d’accouchement à domicile dans les principaux Etats de l’Europe* (Paris: Victor Masson et Fils, 1866).

60 Breisky, ‘Petition des Centralvereins’, 29; Loudon, *Death in Childbirth*, 49.

61 Breisky, ‘Petition des Centralvereins’, 27–9.

62 Ibid., 29.

63 The debates took place in four consecutive meetings of the Supreme Medical Council on 26 July 1879, 22 November 1879, 13 December 1879 and 20 December 1879. ÖStA, AVA, Ministerium des Inneren, Allgemein 36/2, Karton 951, 1879, Zl. 8152.

64 Cf. Attachment to the meeting of the Supreme Medical Council, 13 December 1879. ÖStA, AVA, Ministerium des Inneren, Allgemein 36/2, Karton 951, 1879, Zl. 8152.

65 ‘Verordnung des Ministeriums des Inneren vom 4. Juni 1881, mit welcher eine revidirte Hebammen-Instruction erlassen wird’, *Reichsgesetzblatt für die im Reichsrathe vertretenen Königreiche und Länder* 1881, 54, 212–5. This version did not include the appendix. It can be found in ‘Verordnung des Ministeriums des Inneren vom 4. Juni 1881, mit welcher eine revidirte Hebammen-Instruction erlassen wird’, *Landes-Gesetz- und Verordnungsblatts für das Königreich Galizien und Lodomerien sammt dem Großherzogthume Krakau*, 1881, 49, 119–33.

66 Reinhold Zilch, ‘Das preußische Kultusministerium und die amtlichen Hebammenlehrbücher 1815–1904’, in Daniel Schäfer, ed, *Rheinische Hebammengeschichte im Kontext* (Kassel: kassel university press, 2010), 159–95.

67 Curtis, ‘Midwives and their Role’, 332. Løkke, ‘Antiseptic Transformation’, 112.

68 It is very likely that Scandinavians studying at Austrian universities or young Scandinavian doctors who visited Austrian clinics during their study trips across Europe, experienced the changed obstetric routines and took them home with them, cf. Stephan Curtis, ‘Swedish in Name Only: The International Education of Nineteenth-Century Swedish Medical Students and Practitioners’, *History of Science*, 2012, 1, 257–88.

69 Bohle, *Hebammen*, 74–95.

70 On the ‘practice without theory’-debate and evidence-based appropriation see Bleker, ‘Antisepsis in Deutschland’, 288–94.

71 ÖStA, AVA, Ministerium des Inneren, Allgemein 36/2, Karton 951, 1878, Zl. 10381.

72 ÖStA, AVA, Ministerium des Inneren, Allgemein 36/2, Karton 951, 1879, Zl. 28375, 3699.

73 For more detailed information on the university reform after 1848 see: Christof Aichner, ‘The Reforms of the Austrian University System 1848–1860 and their Influence on the Process of Discipline Formation’, in A Simoes et al., eds, *Sciences in the Universities of Europe, Nineteenth and Twentieth Centuries* (Boston Studies in the Philosophy and History of Science 309) (Dordrecht: Springer Science+Business Media, 2015), 293–309.

74 ÖStA, AVA, Ministerium des Inneren, Allgemein 36/2, Karton 951, 1881, Zl. 3512.

75 Zilch, ‘Das preußische Kultusministerium’, 159–95.

76 ÖStA, AVA, Ministerium des Inneren, Allgemein 36/2, Karton 951, 1879, Zl. 28375, 3699.

77 Josef Späth, *Lehrbuch der Geburtshilfe für Hebammen* (Wien: Wilhelm Braumüller, 1869).

78 The book was reprinted in four editions, dating to 1874,1880 and 1886. Josef Späth, *Lehrbuch der Geburtshilfe für Hebammen*, (Wien: Braumüller ^2^1874, ^3^1880; ^4^1886).

79 Kleinwächter, *Lehrbuch der Hebammenkunst*.

80 Lodovico Kleinwächter, *Compendio di ostetricia ad uso delle levatrici* (Innsbruck: Libreria accademica Wagneriana, 1881).

81 Kleinwächter, *Lehrbuch der Hebammenkunst*, 285–92.

82 Hermann Fehling, ‘Review on Kleinwächter, Lehrbuch der Hebammenkunst, 1879’, *Centralblatt für Gynäkologie*, 1880, 4, 103–4.

83 N.N., *Lehrbuch der Geburtshülfe für die Hebammen* (Berlin: August Hirschwald, 1878), 293.

84 Fehling, ‘Review’, 103–4.

85 Ferdinand Weber-Ebenhof, *Lehrbuch der Geburtshilfe für Hebammen* (Prag: Urbánek, 2nd revised edition 1878). The first edition was published in 1871.

86 Ferdinand Weber-Ebenhof, *Das antiseptische Verfahren in der Geburtshilfe. Ein Leitfaden für Geburtshelferinnen und Belehrung für Frauen* (Prag: Urbánek, 1880).

87 Ferdinand Weber-Ebenhof, *Antiseptické ošetřování rodiček a nedělek. Návod pro babičky, jak mohou novým protihnilobným spůsobem ošetřování zameziti veškerou nákazu rodiček a šestinedělek, a poučení pro matky, jak mohou být ušetřeny před zhoubnými nemocemi šestinedělí* (Prag: Urbánek, 1880).

88 Ferdinand Weber-Ebenhof, *Antiseptické ošetřování rodiček a nedělek : návod pro babičky, jak mohou novým protihnilobným spůsobem ošetřování zameziti veškerou nákazu rodiček a šestinedělek, a poučení pro matky, jak mohou být ušetřeny před zhoubnými nemocemi šestinedělí* (Prag: Urbánek, 1886).

89 Weber-Ebenhof, *Das antiseptische Verfahren*, 3.

90 Gottfried Ritter von Rittershain, ‘Preview on Das antiseptische Verfahren in der Geburtshilfe by Ferdinand Ritter von Weber-Ebenhof, 1880’ *Prager medicinische Wochenschrift*, 1879, 21, 209.

91 ÖStA, AVA, Ministerium des Inneren, Allgemein 36/2, Karton 951, 1878, Zl. 10381; Elena Taddei, *Franz von Ottenthal. Arzt und Tiroler Landtagsabgeordneter (1818–1899)* (Wien: Böhlau, 2010), 168.

92 N.N., ‘Letter to the editors’, *Hebammen-Zeitung*, 1888, 16, 126.

93
*Hebammen-Zeitung*, 1888, 12, 93.

94
*Hebammen-Zeitung*, 1890, 5, 38.

95
*Dienstvorschriften für Hebammen* (Wien: k.k. Hof- und Staatsdruckerei, 1897).

96 Piskaček, ‘Reform’, 500–1; Ekstein, ‘Sozialhygiene des Puerperalfiebers’, 161–5.

97 Bohle, *Hebammen*, 76.

98 N.N., ‘Letter to the editors’, *Hebammen-Zeitung*, 1888, 16, 126.

99 Weber-Ebenhof, *Das antiseptische Verfahren*, titel page.

100
*Ibid*., 4.

101
*Ibid*., 8. Cf. also Ritter, ‘Preview on Das antiseptische Verfahren, 1880’, 209.

102
*Neue Freie Presse* 1878, 5068, 4. Non-compliance was also found for the Swedish example, see Curtis, ‘Midwives and their Role’, 332.

103 Carter, ‘Ignaz Semmelweis’.

104 Kleinwächter, *Lehrbuch der Hebammenkunst*, 289.

105 Späth, *Lehrbuch der Geburtshilfe*, Preface.

106 Kleinwächter, *Lehrbuch der Hebammenkunst*, 287.

107 Weber-Ebenhof, *Das antiseptische Verfahren*, 3–5.

108 Carl Breus, ‘Zur Reform des Hebammenwesens’, *Hebammen-Zeitung*, 1888, 9, 64–7; Emil Ehrendorfer, ‘Ein Mittel zur Verhütung des Wochenbettfiebers’, *Hebammen-Zeitung*, 1889, 16, 124–5; Richard Lumpe, ‘Ueber Antiseptik’, *Hebammen-Zeitung*, 1889, 24, 185–7; Otto Weiß, ‘Ueber Erkrankungen des Wochenbettes’, *Hebammen-Zeitung*, 1890, 2, 11–3.

109 Ekstein, ‘Sozialhygiene des Puerperalfiebers’, 161–5.

110 Kleinwächter, *Lehrbuch der Hebammenkunst*, 286.

111 Weber-Ebenhof, *Das antiseptische Verfahren*, 4.

112 Kleinwächter, *Lehrbuch der Hebammenkunst*, 291.

113 Weber-Ebenhof, *Das antiseptische Verfahren*, 8.

114 Sofie Staudinger, ‘Report’, *Hebammen-Zeitung*, 1887, 2, 6–7.

115 J.R., ‘Letter to the editors’, *Hebammen-Zeitung*, 1888, 19, 150.

116 ÖStA, AVA, Ministerium des Inneren, Allgemein 36/2, Karton 951, 1879, Zl. 8152.

117 Editors’ response to N.N., ‘Letter to the editors’, *Hebammen-Zeitung*, 1890, 1, 6.

118 Ludwig Kleinwächter, ‘Review on Simon Baruch, “The prevention of puerperal infection” 1884’, *Deutsches Archiv für Geschichte der Medicin und medicinische Geographie*, 1885, 8, 231–2; Ludwig Kleinwächter, ‘Die Rückwirkung der aseptischen Gynäkologie und Geburtshilfe auf die Pathologie’, *Wiener Medizinische Presse. Organ für praktische Aerzte*, 1894, 27, 1041–4; Ludwig Kleinwächter, ‘Über die im Verlaufe der letzten Jahre zu verzeichnenden Fortschritte in der Lehre vom Puerperalfieber’, *Wiener Klinik: Vorträge aus der gesamten Praktischen Heilkunde*, 1906, 32, 343–98. Ehrendorfer, ‘Behandlung’, 179–94.

119 Weber-Ebenhof, *Das antiseptische Verfahren*, 15–6.

120
*Ibid*., 20.

121 Kleinwächter, *Lehrbuch der Hebammenkunst*, 290. Weber-Ebenhof, *Das antiseptische Verfahren*, 21.

122 Weber-Ebenhof, *Das antiseptische Verfahren*, 19.

123
*Ibid*., 10.

124
*Ibid*., 12–3.

125 Kleinwächter, *Lehrbuch der Hebammenkunst*, 291.

126
*Ibid*., 288.

127
*Ibid*., 289.

128
*Ibid*., appendix; Weber-Ebenhof, *Das antiseptische Verfahren*, 23.

129 Louise Wallner, ‘Ein Beitrag zur Antiseptik am Lande!’, *Hebammen-Zeitung*, 1890, 9, 70.

130 ÖStA, AVA, Ministerium des Inneren, Allgemein 36/2, Karton 951, 1881, Zl. 3512.

131
*Ibid*., 8152.

132 Weber-Ebenhof, *Das antiseptische Verfahren*, 8 and 24–6; Otto Weiß, ‘Ueber Desinfectionsmittel’, *Hebammen-Zeitung*, 1888, 20–1, 153–4 and 161–3.

133 ÖStA, AVA, Ministerium des Inneren, Allgemein 36/2, Karton 951, 1879, Zl. 28375, 3669.

134 Weiß, ‘Ueber Desinfectionsmittel’, 21, 161–2.

135
*Hebammen-Zeitung*, 1887, 5, 6; *Hebammen-Zeitung*, 1889, 11, 86; *Hebammen-Zeitung*, 1890, 1, 6.

136 Curtis, ‘Midwives and their Role’, 332.

137 B.W., ‘Letter to the editors’, *Hebammen-Zeitung*, 1889, 10, 78; M.D., ‘Letter to the editors’, *Hebammen-Zeitung*, 1890, 15, 118–9.

138 Hubert Peters, ‘Ueber Ausspülungen der Scheide und der Gebärmutter während der Fortpflanzungsperiode des Weibes’, *Hebammen-Zeitung*, 1887, 5, 1–3; Hans Koffer, ‘Ueber Kindbettfieber’, *Hebammen-Zeitung*, 1890, 20, 153–5.

139 Therese Kiridus, ‘Letter to the editors’, *Hebammen-Zeitung*, 1888, 17, 133–4; M.D., ‘Letter to the editors’, 119.

140 Piskaček, ‘Reform’, 498–502.

141 ‘Regelung des Hebammenwesens’, *Bundesgesetzblatt der Republik Österreich*, 1925, 214, 834–7; ‘Dienstordnung für Hebammen’, *Bundesgesetzblatt der Republik Österreich*, 1929, 21, 166–84.

## Notes

Marina Hilber is an Assistant Professor of Social and Economic History at the University of Innsbruck. She is co-editor of the medical historical journal *VIRUS. Beiträge zur Sozialgeschichte der Medizin*. Her current research focuses on the social history of childbirth, the science history of obstetrics and midwifery, as well as the social history of polio in Austria. This article is based on her research project ‘Medical Networks. Ludwig Kleinwächter’s (1839–1906) Participation in the Transnational Scholarly Network of Gynaecology and Obstetrics’, funded by the Austrian Science Fund (FWF) [T 871-G28, 2017–2021].

